# Association between loneliness and incident use of benzodiazepines and opioids: analyses of the Canadian Longitudinal Study on Aging

**DOI:** 10.1093/ageing/afag267

**Published:** 2026-09-14

**Authors:** Jennifer A Watt, Shristi Sharma, Yu Qing Huang, Aaron Jones, Kathryn Nicholson, Amy Y X Yu, Sharon Straus, Michael Tyler Dipaolo, Moira K Kapral, Manav V Vyas

**Affiliations:** St. Michael’s Hospital-Unity Health Toronto, Toronto, Canada; Division of Geriatric Medicine, University of Toronto, Toronto, Canada; St. Michael’s Hospital-Unity Health Toronto, Toronto, Canada; Department of Epidemiology and Biostatistics—Epidemiology & Biostatistics, Schulich School of Medicine and Dentistry, London, Ontario, Canada; St. Michael’s Hospital-Unity Health Toronto, Toronto, Canada; Division of Geriatric Medicine, University of Toronto, Toronto, Canada; Department of Health Research Methods, Evidence, and Impact, McMaster University, Hamilton, Ontario, Canada; Department of Epidemiology and Biostatistics—Epidemiology & Biostatistics, Schulich School of Medicine and Dentistry, London, Ontario, Canada; Department of Medicine (Neurology), University of Toronto, Sunnybrook Health Sciences Centre, Toronto, ON, Canada; Division of Geriatric Medicine, University of Toronto—Medicine, Toronto, Ontario, Canada; Knowledge Translation Program, St Michael’s Hospital Li Ka Shing Knowledge Institute, Toronto, Ontario, Canada; The Royal College of Surgeons in Ireland -Bahrain, Manama, Bahrain; Department of Medicine, University of Toronto, Toronto, Ontario, Canada; St. Michael’s Hospital-Unity Health Toronto, Toronto, Canada; Division of Neurology, University of Toronto, Toronto, Canada

**Keywords:** CLSA, loneliness, benzodiazepine, opioid, medications, older people

## Abstract

**Background:**

The association between loneliness and incident use of benzodiazepines and opioids, and sociodemographic factors associated with their use, is unknown.

**Methods:**

Using the Canadian Longitudinal Study on Aging’s comprehensive cohort, we conducted a longitudinal study to understand how loneliness was associated with incident use of benzodiazepines and opioids, separately, using multivariable modified logistic regression models. We evaluated sociodemographic factors associated with benzodiazepine and opioid use among lonely participants.

**Results:**

We included 29 976 participants, of whom 10.8% were lonely (all the time or occasionally) at baseline. Lonely participants were older [mean age 64.4, standard deviation (SD) 10.5 vs. mean age 62.8, SD 10.2] and more likely to be female (57.9% vs. 50.1%) and live alone (45.2% vs. 19.9%) than participants who were not lonely. Lonely participants had a higher rate of incident use of benzodiazepines [73 (2.8%) vs. 305 (1.3%); adjusted odds ratio (aOR) 1.94, 95% confidence interval (95% CI) 1.47–2.57] and opioids [60 (2.2%) vs. 283 (1.2%); aOR 1.43, 95% CI 1.05–1.93] compared to those who were not lonely. Loneliness remained significantly associated with use of benzodiazepines, but not opioids, after adjusting for mood and anxiety disorders. Lonely females and non-Whites were more likely to be initiated on benzodiazepines, whereas lonely immigrants were less likely to be initiated on benzodiazepines.

**Conclusions:**

Loneliness was associated with incident use of benzodiazepines and opioids. Multimodal interventions to deprescribe these medications in lonely individuals and decrease symptoms of loneliness could mitigate their attendant health effects.

## Key points

- Loneliness was associated with incident use of benzodiazepines and opioids in Canadian adults.
- This association persisted for benzodiazepines even after adjusting for mood and anxiety disorders, but not for opioids.
- Females and non-White participants higher odds, and immigrants had lower odds of incident benzodiazepine use.

## Introduction

Benzodiazepines and opioids are among the most commonly prescribed potentially inappropriate medications in older adults [1, 2]. Despite initiatives to improve the appropriateness of benzodiazepine and opioid prescribing because of their association with adverse drug events, including an increased risk of falls, cognitive decline and mortality, prescribing trends remain problematic [1–5]. In England, 11% of adults aged 60 to 69 years old were prescribed an opioid in 2022 [6]. In the UK, 2.7% of adults over 65 years were prescribed a benzodiazepine in 2018 [7]. However, there is substantial variation in prescribing across countries [7–9].

More than 12% of adults aged 65 years or older in England report feeling lonely at least some of the time [10]. Loneliness is a subjective, unwelcome feeling of a lack of or loss of companionship that occurs when there is a mismatch between desired and perceived quantity and quality of social relationships [11]. There is growing concern about increased risks of potentially inappropriate prescribing in this population [12]. Lonely individuals are more likely to have anxiety and mood disorders, for which benzodiazepines may be used [13, 14]. Loneliness is also associated with a higher rate of chronic pain, with an even higher risk in those with co-existing anxiety and depression, potentially leading to opioid use [15, 16]. Loneliness has been associated with daily use of benzodiazepines and opioids, but not non-opioid analgesics, in a cross-sectional study [15]. Similarly, loneliness has been associated with polypharmacy in females in a cross-sectional study [17]. In contrast, living alone, a known risk factor for loneliness, has not been associated with prescription use of benzodiazepines and opioids [18]. The temporal association between loneliness and incident use of these medications has not yet been evaluated, nor has the role of anxiety and mood disorders been considered, limiting our ability to draw a causal link. Understanding this association could have important implications for how we assess and manage loneliness and appropriate prescribing.

Using data from the Canadian Longitudinal Study on Aging (CLSA), we conducted a longitudinal cohort analysis to evaluate the association between loneliness and incident benzodiazepine and opioid use, and whether it attenuated after adjusting for anxiety and mood disorders. We hypothesised that loneliness would be associated with an increased risk of incident use of these medications, and this increased risk would be attenuated after accounting for mood disorders and anxiety.

## Methods

We reported this longitudinal cohort study as per the Strengthening the Reporting of Observational Studies in Epidemiology Statement [19].

### Ethics

This study was approved by St. Michael’s Hospital-Unity Health Toronto REB (REB # 21262).

### Cohort

**Figure 1:**
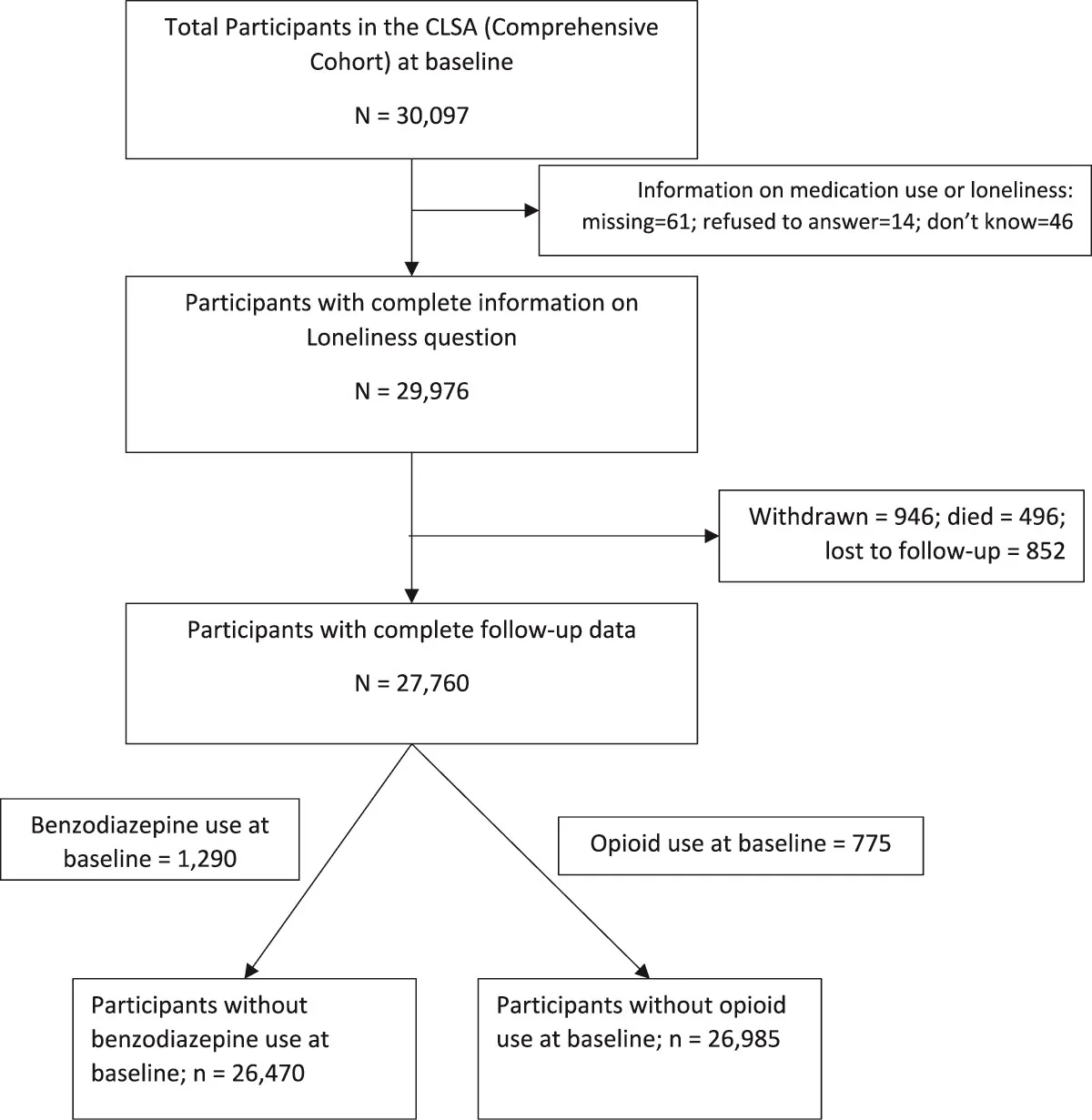
Flow diagram of the included participants.

The CLSA is a longitudinal cohort study that enrolled 51 338 community-dwelling Canadian adults aged 45 to 85 years at baseline (2011–2015) [20]. We analysed data from the CLSA comprehensive cohort (*n* = 30 097) because medication data are collected by trained interviewers only in this cohort. We determined the use of benzodiazepines and opioids at baseline (2011–2015) and at the follow-up assessment (2015–2018). We excluded those who were missing data on medication use (either missing, refused to answer or answered don’t know), those who withdrew during follow-up, and those who died during follow-up (Figure 1). For our primary outcome of incident use of benzodiazepines or opioids at follow-up, we further excluded those using these medications at baseline.

**Figure 2:**
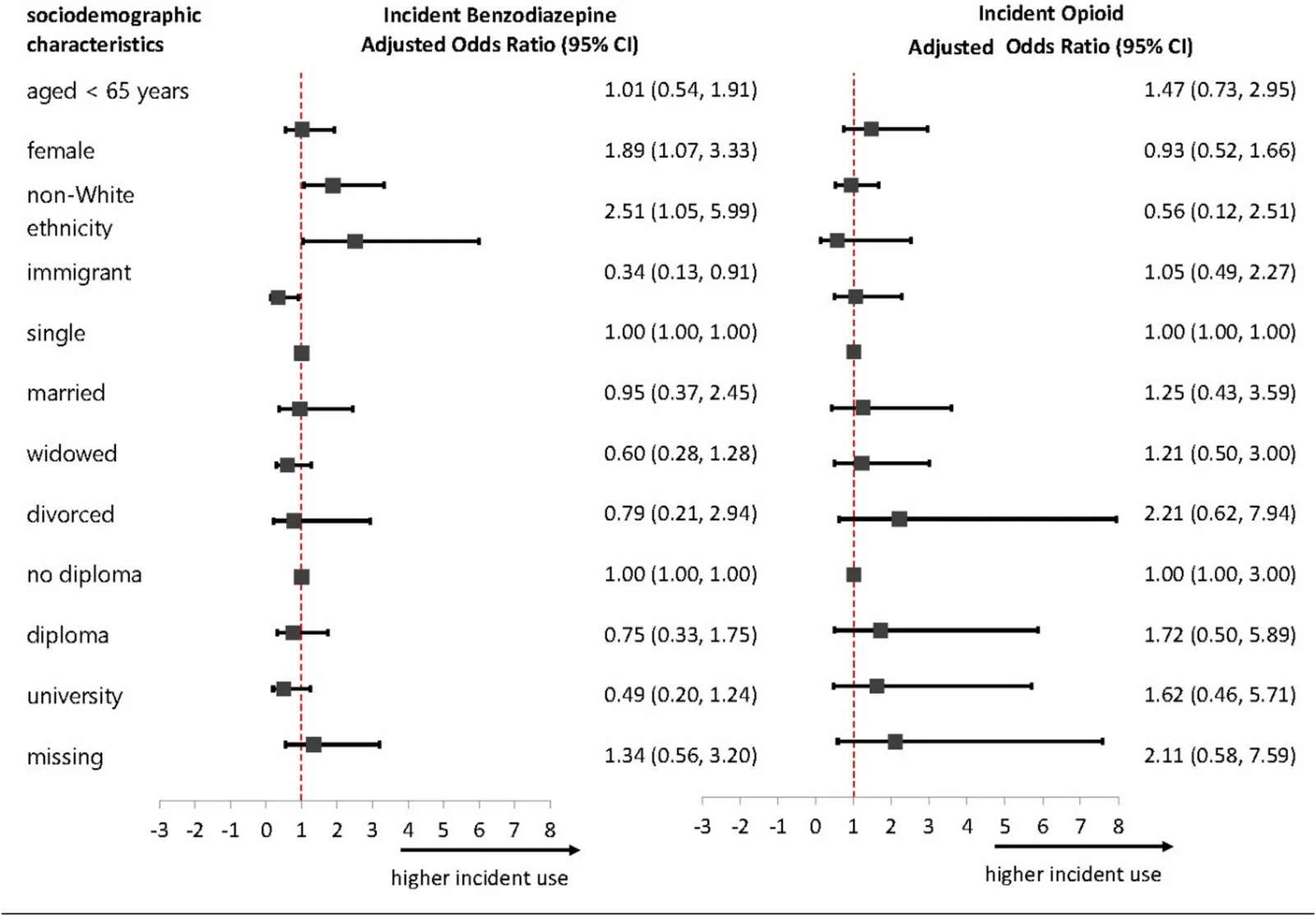
Association between sociodemographic factors and incident use of benzodiazepines (*n* = 2608) and opioids (*n* = 2735) among lonely adults. Odds ratios are additionally adjusted for functional status, living alone status, employment status, smoking status, alcohol use, and comorbidities (hypertension, diabetes, osteoarthritis, cancer, heart disease and stroke) and mood and anxiety disorders. n, number.

### Exposures

We obtained information on loneliness at baseline and follow-up using the Centre for Epidemiologic Studies (CES-D) depression scale [21]. Participants were asked to rate loneliness in the past week using the question: ‘How often did you feel lonely?’ Response options included: 1 = all the time (5–7 days); 2 = occasionally (3–4 days); 3 = some of the time (1–2 days) and 4 = rarely or never (less than 1 day). We classified those who responded ‘all the time’ or ‘occasionally’ as *lonely* [22]. This definition has a specificity of 85% when compared to the 3-item University of Los Angeles Loneliness scale [23].

We ascertained the history of self-reported anxiety disorder at baseline from an affirmative response to the question ‘Has a doctor ever told you that you have an anxiety disorder, such as a phobia, obsessive-compulsive disorder, or panic disorder?’ We ascertained the history of self-reported mood disorder at baseline from an affirmative response to the yes/no question ‘Has a doctor ever told you that you have a mood disorder such as depression (including manic depression), bipolar disorder, mania, or dysthymia?’ The selection of covariates was based on our previous work, clinical expertise and literature [24], and we modelled covariates as per the methods applied in previous studies analysing CLSA data [12, 22]. We modelled covariates as continuous, binary, ordinal or categorical, as indicated in Table 1.

**Table 1:** Baseline characteristics of people with complete baseline data in CLSA comprehensive cohort (*N* = 27 760).

Characteristics of interest	Lonely *N* = 3245	Not lonely *N* = 26 731	Standardised difference
Female, *n* (%)	1880 (57.9)	13 385 (50.1)	0.158
Mean age in years (SD)	64.4 (10.5)	62.8 (10.2)	0.157
Ethnicity, *n* (%)			
White	3003 (92.5)	25 264 (94.5)	0.08
Black	28 (0.9)	190 (0.7)	0.017
Asian	104 (3.2)	641 (2.4)	0.049
Others	33 (1.0)	239 (0.9)	0.013
Missing	77 (2.4)	397 (1.5)	0.065
Immigrant, *n* (%)	589 (18.2)	4820 (18.0)	0.003
Marital status, *n* (%)			
Single	469 (14.5)	2176 (8.1)	0.201
Married or common-law	1344 (41.4)	19 254 (72.0)	0.650
Widowed	1279 (39.4)	4659 (17.4)	0.502
Divorced/Separated	153 (4.7)	634 (2.4)	0.127
Missing	0 (0.0)	8 (0.0)	0.024
Education level, *n* (%)			
No diploma	283 (8.7)	1948 (7.3)	0.053
Diploma/certificate	1170 (36.1)	8521 (31.9)	0.088
University and higher	1151 (35.5)	12 374 (46.3)	0.221
Others	0 (0.0)	38 (0.1)	0.053
Missing	641 (19.8)	3850 (14.4)	0.143
Living alone, *n* (%)	1467 (45.2)	5310 (19.9)	0.562
Employment status, *n* (%)			
Not employed	284 (8.8)	1133 (4.2)	0.184
Working	1008 (31.1)	10 874 (40.7)	0.202
Retired	1953 (60.2)	14 717 (55.1)	0.104
Functional impairment, *n* (%)			
None	2681 (82.6)	24 320 (91.0)	0.249
Mild	490 (15.1)	2144 (8.0)	0.222
Moderate/Severe/Total	56 (1.7)	185 (0.7)	0.095
Missing	18 (0.6)	82 (0.3)	0.038
Smoking status, *n* (%)			
Current	457 (14.1)	2240 (8.4)	0.181
Never	1370 (42.2)	12 817 (48.0)	0.115
Former	1418 (43.7)	11 673 (43.7)	0.001
Alcohol use, *n* (%)			
≥4 times/month	1566 (48.3)	15 651 (58.6)	0.207
Less than 4 times/month	1033 (31.8)	7599 (28.4)	0.074
Never	537 (16.6)	2865 (10.7)	0.171
Missing	109 (3.4)	616 (2.3)	0.064
Comorbidities, *n* (%)			
Mood	983 (30.3)	4149 (15.5)	0.357
Anxiety	531 (16.4)	2060 (7.7)	0.269
Osteoarthritis	1057 (32.6)	6852 (25.6)	0.153
Any cancer	352 (10.8)	2650 (9.9)	0.031
Diabetes	698 (21.5)	4596 (17.2)	0.111
Hypertension	1354 (41.7)	9723 (36.4)	0.109
Heart disease	547 (16.9)	3521 (13.2)	0.103
Stroke	203 (6.3)	1136 (4.2)	0.094

### Outcomes

The primary outcome was incident benzodiazepine or opioid use at follow-up, and the secondary outcome was prevalent use of these medications at baseline. Benzodiazepine or opioid use was defined as the use of one or more of these medications, taken orally (prescription and non-prescription). We identified these medications using Drug Identification Number (DIN) lists obtained from Health Canada’s website for approved medications containing opioids or benzodiazepines. Medication data were entered using the DIN, which is a unique number assigned to medications approved by Health Canada. Participants were asked to show the trained interviewer all regularly or occasionally used medications, including prescription and nonprescription medications. We excluded people who were lost to follow-up, withdrew or died during follow-up.

### Statistical analyses

We compared characteristics of lonely individuals to those of individuals who were not lonely at baseline using standardised differences, with a value of 0.10 or greater suggestive of meaningful differences [25]. We reported the number and proportion of participants with prevalent benzodiazepine and opioid use and, among those who never used these medications, the incident use of these medications at follow-up. We completed all analyses in SAS Enterprise Guide version 9.0 (SAS Institute Inc.).

#### Cross-sectional analyses

We constructed a set of logistic regression models for our outcomes: Model A = age- and sex-adjusted; Model B = covariate-adjusted model (i.e. Model A + covariates listed in Table 1 above, except mood and anxiety disorders) and Model C = fully-adjusted model (i.e. Model B + anxiety and mood disorders). We calculated adjusted odds ratios (aOR) of [1] benzodiazepine use at baseline and [2] opioid use at baseline in logistic regression models, comparing the odds in participants who were lonely to those who were not lonely. Model assumptions were evaluated; the logistic regression models met all model assumptions.

#### Analyses testing for a temporal association

We applied the same models as above, but with the outcome of incident use of benzodiazepines and opioids at follow-up (i.e. people who used benzodiazepines and opioids at baseline were excluded). To evaluate if there were any sex differences in temporal associations in our outcomes, we conducted sex-stratified analyses.

To understand who among lonely adults was at risk of using these medications, we limited our sample to lonely adults and constructed logistic regression models with all covariates as described in Table 1 to describe the adjusted risk ratio of incident use of benzodiazepines and opioids across different social demographic factors.

#### Sensitivity analyses

To test for a reverse association, we evaluated whether use of benzodiazepines and opioids at baseline would be associated with incident loneliness at follow-up, using multivariable models (Model C). Given that the presence of loneliness is not static, we excluded individuals whose loneliness status changed at follow-up (in either direction) to evaluate the association between loneliness and incident drug use in these individuals. To account for variable follow-up times between the baseline and follow-up survey completion in our cohort participants (Supplementary Figure S1), we ran multivariable Poisson models (Model C) with a person-time (or follow-up time) offset.

## Results

We included 29 976 individuals who had complete information on our exposure of interest at baseline (Figure 1), 3245 (10.8%) of whom were lonely at baseline. Compared to those who were not lonely, lonely participants were more likely to be older [mean age 64.4, standard deviation (SD) 10.5 vs. mean age 62.8, SD 10.2], female (57.9% vs. 50.1%) and live alone (45.2% vs. 19.9%) (Table 1).

### Cross-sectional analyses

At baseline, loneliness was associated with higher odds of being a benzodiazepine user [9.6% vs. 4.1%; aOR 1.68, 95% confidence interval (95% CI) 1.45–1.95] and higher odds of being an opioid user (5.2% vs. 2.4%; aOR 1.36, 95% CI 1.12–1.66) in multivariable-adjusted models that did not include a history of mood and anxiety disorders (Model B). However, these associations attenuated, but remained significant, even after adjusting for mood and anxiety disorders (Table 2).

**Table 2:** Cross-sectional and temporal association between loneliness and incident benzodiazepine and opioid use.

	Lonely vs. not lonely, *n* (%)	MODEL A Age-and sex-adjusted OR (95% CI)	MODEL B Age- + sex- + covariate^a^-adjusted OR (95% CI)	MODEL C Age- + sex- + covariate^a^- + mood & anxiety disorder- adjusted OR (95% CI)
Cross-sectional analyses				
Prevalent use of benzodiazepines	310 (9.6) vs. 1092 (4.1)	*n* = 29 976	*n* = 28 920 (96.5%)	*n* = 28 845 (96.2%)
		2.29 (2.00–2.61)	1.68 (1.45–1.95)	1.36 (1.17–1.58)
Prevalent use of opioids	169 (5.2) vs. 646 (2.4)	*n* = 29 976	*n* = 28 920 (96.5%)	*n* = 28 845 (96.2%)
		2.14 (1.80–2.55)	1.36 (1.12–1.66)	1.24 (1.02–1.51)
Longitudinal analyses				
Incident use of benzodiazepines	73 (2.8) vs. 305 (1.3)	*n* = 26 470	*n* = 25 600 (96.7%)	*n* = 25 543 (96.5%)
		2.06 (1.59–2.07)	1.94 (1.47–2.57)	1.63 (1.23–2.17)
Incident use of opioids	60 (2.2) vs. 283 (1.2)	*n* = 26 985	*n* = 26 096 (96.7%)	*n* = 26 031 (96.5%)
		1.81 (1.36–2.40)	1.43 (1.05–1.93)	1.34 (0.99–1.83)

**Prevalent use**—use of medications at baseline, **Incident use**—new use of medications during follow-up. *n*, number.

^a^ **Covariates**—ethnicity, immigration status, marital status, employment status, living alone status, functional status, smoking status, alcohol use and comorbidities (hypertension, diabetes, osteoarthritis, cancer, heart disease and stroke).

### Analyses testing for temporal associations

After excluding individuals who used benzodiazepines at baseline (*n* = 1200), loneliness was associated with higher adjusted odds of incident benzodiazepine use at follow-up (2.8% vs. 1.3%; aOR 1.94, 95% CI 1.47–2.57) in multivariable models that did not include a history of mood and anxiety disorders (Model B). Similarly, after excluding those who used opioids at baseline (*n* = 685), loneliness was associated with higher adjusted odds of incident opioid use at follow-up in multivariable models that did not include a history of mood and anxiety disorders (Model B) (2.2% vs. 1.2%; aOR 1.43, 95% CI 1.05–1.93) (Table 2). However, after adjusting for mood and anxiety disorders, the strength of this association was attenuated such that it was no longer significant for incident opioid use (aOR 1.34, 95% CI 0.99–1.83), but remained statistically significant for benzodiazepine use (aOR 1.63, 95% CI 1.23–2.17) (Table 2). No significant differences were noted between the sexes (results not shown).

Among lonely adults who did not use benzodiazepines (*n* = 2608) or opioids (*n* = 2735) at baseline, female sex (aOR 1.89, 95% CI 1.07–3.33) and non-White ethnicity (aOR 2.51, 95% CI 1.05–5.99) were associated with higher odds of incident benzodiazepine use, while being an immigrant was associated with lower odds of incident benzodiazepine use (aOR 0.34, 95% CI 0.13–0.91), after adjusting for all variables listed in Table 1; however, no sociodemographic factors were associated with incident opioid use (Figure 2). The association between loneliness and incident co-use of benzodiazepines and opioids was not possible, as only 13 participants were incident co-users in the entire cohort.

### Sensitivity analyses

Compared to individuals who did not use benzodiazepines at baseline, those who did had similar odds of becoming lonely at follow-up (13.4% vs. 6.5%, aOR 1.20; 0.97–1.50). Compared to individuals who did not use opioids at baseline, individuals who did had higher odds of becoming lonely at follow-up (15.5% vs. 7.0%, aOR 1.47; 1.12–1.93). Upon excluding 3265 (12.8%) individuals whose loneliness status changed at follow-up, the association between loneliness and incident benzodiazepine use persisted (aOR 2.32; 1.53–3.50). Upon excluding 3369 (13.0%) individuals whose loneliness status changed at follow-up, the association between loneliness and incident opioid use remained non-significant (aOR 1.44; 0.88–2.34). In multivariable Poisson models accounting for variable follow-up, baseline benzodiazepine use (adjusted incidence rate ratio 1.59; 1.20–2.09), but not opioid use (adjusted incidence rate ratio 1.33; 0.98–1.80), was significantly associated with incident loneliness.

## Discussion

In this longitudinal cohort study, we found that participants who were lonely at baseline had a 1.2 to 1.5-fold higher odds of becoming a new user of benzodiazepines and opioids at follow-up, respectively. While the observed associations attenuated after adjusting for anxiety and mood disorders, suggesting their role as potential mediators of these associations, the elevated odds of incident benzodiazepine use in lonely adults persisted. Among lonely adults, being female and of non-White ethnicity were independently associated with higher odds of benzodiazepine use at follow-up, while being an immigrant was associated with lower odds of benzodiazepine use at follow-up. There was no association between sociodemographic factors and incident opioid use.

Our finding of a temporal association between loneliness and incident use of benzodiazepines and opioids, independent of age and co-occurring conditions, further supports the growing literature identifying the negative effects associated with loneliness [26–29]. Potential reasons for the observed associations between loneliness and benzodiazepine or opioid use include amplification of anxiety, pain perception and distress in lonely adults, coupled with increased reliance on pharmacological means to manage symptoms of distress rather than nonpharmacological support derived from social networks [30, 31]. It is also possible that a lonely adult seen by a healthcare provider is misperceived to have physical symptoms of depression or pain, and hence, be prescribed these medications. The elevated risk of falls associated with loneliness may, in part, be related to the adverse use of these medications, which are known risk factors for falls in older adults [5, 32]. Future work using longitudinal studies should evaluate the impact of these medications on the association between loneliness and falls.

The attenuation in the observed associations between loneliness and the use of these medications is in line with current clinical practice, whereby people with anxiety or mood disorders may be prescribed benzodiazepines [33, 34]. However, in contrast to this hypothesis, we found that attenuation in incident opioid use was greater and the association between loneliness and incident opioid use became non-significant. One potential reason for this may be the small number of incident opioid users in our sample. Another possibility is that public health policies have primarily focused on opioid misuse compared to benzodiazepine misuse [35]. For example, in Canada, a federally-regulated, uniform risk warning for opioids was introduced in 2018; in contrast, a stronger, standardised warning for benzodiazepines was introduced in 2020, but without the need for a physical sticker. We observed that a very small number of participants used these medications together, limiting our ability to evaluate the association between loneliness and co-use of these medications. Use of these medications together has been associated with an even higher risk of falls and mortality [36, 37], and public health policies have discouraged their co-use. That the use of benzodiazepines at baseline was not associated with incident loneliness, unlike the baseline use of opioids, further supports the temporal association between loneliness and incident benzodiazepine use, and provides some support for this being causal in nature.

Our findings have important implications for patients, family or friend carers, clinicians and policymakers. If patients are being assessed for potentially inappropriate prescribing of benzodiazepines, they should be assessed for the presence of loneliness as well as mood and anxiety disorders. If loneliness is detected, clinicians, patients and family or friend carers can engage in shared decision-making to select among evidence-based nonpharmacologic interventions to decrease loneliness, such as group-based interventions (e.g. cognitive behavioural therapy, mindfulness, exercise) or internet training (e.g. how to use social media or email) [38]. Offering of these nonpharmacologic interventions targeting symptoms of loneliness can be offered alongside deprescribing of benzodiazepines [39]. Policymakers can support the development of educational opportunities for clinicians to learn about the link between benzodiazepine use and loneliness, as well as evidence-based approaches that support assessment and management [40]. Policymakers can also facilitate access to nonpharmacologic interventions by developing social prescribing and community navigation programs.

Our study has limitations. First, we used the CES-D subscale to ascertain loneliness, whose validity is not as high as some other scales; however, a recent study found good correlation between the three-item University of California, Los Angeles (UCLA) loneliness scale and the CES-D subscale using data from the CLSA (kappa 80%, *P* < .001) [23, 41]. A medication recorded by CLSA staff does not mean that the medication was actually taken, and there is evidence that suggests that loneliness may be associated with poorer medication adherence [42–44]. We did not perform causal mediation analyses to evaluate the mediating effects of mood or anxiety. Future work can consider this approach to describe the direct and indirect mediating effects of mood, anxiety or other mediators [45]. We could not evaluate the association between loneliness and Z-medications (e.g. zopiclone), which are associated with similar negative health impacts as benzodiazepines [46, 47]. Finally, we could not evaluate whether the use of the medication was on an ‘as-needed basis’ or there was overdose, nor could we evaluate the cumulative dose of benzodiazepine or opioids, which is important because a higher cumulative dose is associated with higher rates of falls and mortality [2]. Finally, for some patients, the use of these medications may indeed be justified based on the clinical scenario or reasonable indication (e.g. opioid use at the end of life or for cancer-related pain); however, our data do not allow us to identify whether the use of the medication was justified or not.

In summary, loneliness is temporally associated with incident use of benzodiazepines and opioids. Interventions directed at deprescribing these medications should also address loneliness and co-existing anxiety and mood disorders.

## Supplementary Material

aa-26-1225-File002_afaf267
