# Supplementary material for: Association between loneliness and incident use of benzodiazepines and opioids: analyses of the Canadian Longitudinal Study on Aging
Source: Age Ageing. 2026 Sep 14;55(9):afag267. doi: 10.1093/ageing/afag267 (PMC13574281; doi:10.1093/ageing/afag267)
Supplement: Supplementary file 1 [file aa-26-1225-file002_afaf267.docx]

**Figure S1.** Distribution of follow-up time measured as the difference between the baseline survey and follow-up survey in the CLSA comprehensive cohort (mean = 1074 days, SD 118 days; median =1065 days; Q1-Q3 = 1011-1114 days)
